# Higher plasma aldosterone is associated with increased risk of cardiovascular events in hypertensive patients with suspected OSA: UROSAH data

**DOI:** 10.3389/fendo.2022.1017177

**Published:** 2022-10-07

**Authors:** Lin Gan, Nanfang Li, Mulalibieke Heizhati, Mengyue Lin, Qing Zhu, Xiaoguang Yao, Ting Wu, Menghui Wang, Qin Luo, Delian Zhang, Wen Jiang, Junli Hu

**Affiliations:** Hypertension Center of People’s Hospital of Xinjiang Uygur Autonomous Region, Xinjiang Hypertension Institute, National Health Committee Key Laboratory of Hypertension Clinical Research, Key Laboratory of Xinjiang Uygur Autonomous Region, Hypertension Research Laboratory, Xinjiang Clinical Medical Research Center for Hypertension (Cardio-Cerebrovascular) Diseases, Urumqi, China

**Keywords:** aldosterone, cardiovascular disease, all-cause mortality, hypertension, renin suppression

## Abstract

**Objective:**

To evaluate the association of plasma aldosterone concentration (PAC) with incident cardiovascular disease (CVD) and all-cause mortality in hypertensive patients with suspected obstructive sleep apnea (OSA) and calculate the optimal cut-off value of PAC for this specific population.

**Patients and methods:**

Participants with PAC at baseline in UROSAH in 2011-2013 were enrolled and followed up till 2021. Composite outcome included CVD and all-cause mortality. Cox proportional hazards model was used to evaluate the relationship between PAC and the composite outcome. Time-dependent ROC curve was used to determine the optimal cut-off value of PAC. Besides, we conducted subgroup analyses and sensitivity analyses.

**Results:**

3173 hypertensive participants aged 18-84 years comprised analytical sample. During a median follow-up of 7.3 years and 22640 person-years, 69 deaths and 343 cases of incident CVD occurred. The incidence of composite outcome was increased with elevation in tertile of PAC. Compared with the first tertile, the risk of CVD and all-cause death was higher in third tertile (HR=1.81, 95%CI: 1.39-2.35, P<0.001). Time-dependent ROC curve showed optimal threshold for PAC was 12.5ng/dl. Whether renin was suppressed or not (≤0.5 or >0.5ng/ml per h), elevated PAC was associated with an increased risk of CVD. Our results remained stable and consistent in sensitivity analyses.

**Conclusion:**

Higher PAC was associated with increased risk of CVD and all-cause mortality in hypertensives with suspected OSA, even in the absence of primary aldosteronism (PA). Hypertensives with PAC≥12.5ng/dl showed a significantly increased risk of CVD, indicating that special attention and treatment were required in this specific population.

## Introduction

Cardiovascular disease (CVD) is the leading cause of death and disability-adjusted life years globally, causing one-third of deaths (1, 2). Hypertension is the leading modifiable risk factor for CVD, affecting more than 30% of adults worldwide (3, 4). Due to ageing of the population and lifestyle-related risk factors, burdens of both hypertension and CVD have been increasing. In addition, management of traditional risk factors does not seem to reduce CVD morbidity and mortality, therefore exploring new potential risk factors, such as increased aldosterone levels, may improve the risk prediction of CVD and provide new targets for prevention and or treatment to reduce CVD deaths.

Aldosterone, a key hormone in renin-angiotensin-aldosterone system (RAAS), plays a pivotal role in maintaining homeostasis of fluid and regulation of blood pressure. Long-term activation of the RAAS can lead to excessive PAC, thereby promoting and perpetuating hypertension, congestive heart failure, chronic kidney disease, and metabolic disease (5). Furthermore, excessive PAC is closely related to an increase in event and mortality of CVD (6, 7). In experimental models, excessive circulating and tissue aldosterone have been found to have adverse effects on cardiovascular system through some mechanisms, such as oxidative stress, endothelial dysfunction, inflammation, and interstitial fibrosis (8–10).

Several population-based studies observed that PAC is positively correlated with blood pressure, waist circumference, insulin resistance and other risk factors of CVD (11–14). PAC is also associated with markers of subclinical atherosclerosis including coronary artery calcification and ankle-brachial index (15, 16). However, the reported effects of PAC on CVD and mortality are not always consistent in epidemiological or clinical studies. Some studies have found that elevated PAC leads to development of CVD and all-cause mortality (17, 18), whereas others report that PAC is not associated with CVD (19, 20). In addition, the MESA study has found serum aldosterone concentration is associated with increased risk of all-cause mortality when renin was suppressed (15). The inconsistencies may be, in part, explained by instability of PAC, which can be affected by medications, salt intake, disease state and or ethnicity (21–23). In addition, there is a paucity of study on the relationship between PAC and CVD and all-cause mortality in hypertensive patients. Therefore, to clarify the association of PAC and cardiovascular events and the optimal threshold of PAC in hypertensive patients, we conducted this longitudinal study among hypertensive patients with suspected OSA.

## Materials and methods

### Study population

Participants for current study were the hypertensive patients with PAC measurement at baseline in Urumqi Research on Sleep Apnea and Hypertension (UROSAH).

UROSAH is a single-center observational study to assess the association of OSA with long term cardiovascular outcomes in patients with hypertension. Hypertensive patients aged 18 years and over who visited Hypertension Center of People’s Hospital of Xinjiang Uygur Autonomous Region China between Jan 2011 and Dec 2013 were reviewed. Inclusion criteria of UROSAH were following: hypertensive patients who were suspected of OSA. Exclusion criteria in the present study were as follows: 1. patients with acute severe cardiovascular and cerebrovascular diseases in recent 3 months of enrollment; 2. patients with acute asthma, chronic obstructive pulmonary disease, interstitial lung disease, pulmonary tuberculosis and other respiratory diseases; 3. patients who were using steroids, bronchodilators and antihistamines at the time of hospitalization; 4. patients with malignant tumor, acute infection and autoimmune diseases; 5. renal and renovascular hypertension, pheochromocytoma, Cushing’s syndrome and other common secondary hypertension; 6. patients with failure to sleep study. In total, 3605 hypertensive patients with suspected OSA were included. In addition, patients who were lost to follow-up and without PAC data at baseline were excluded from the current study.

**Figure 1:**
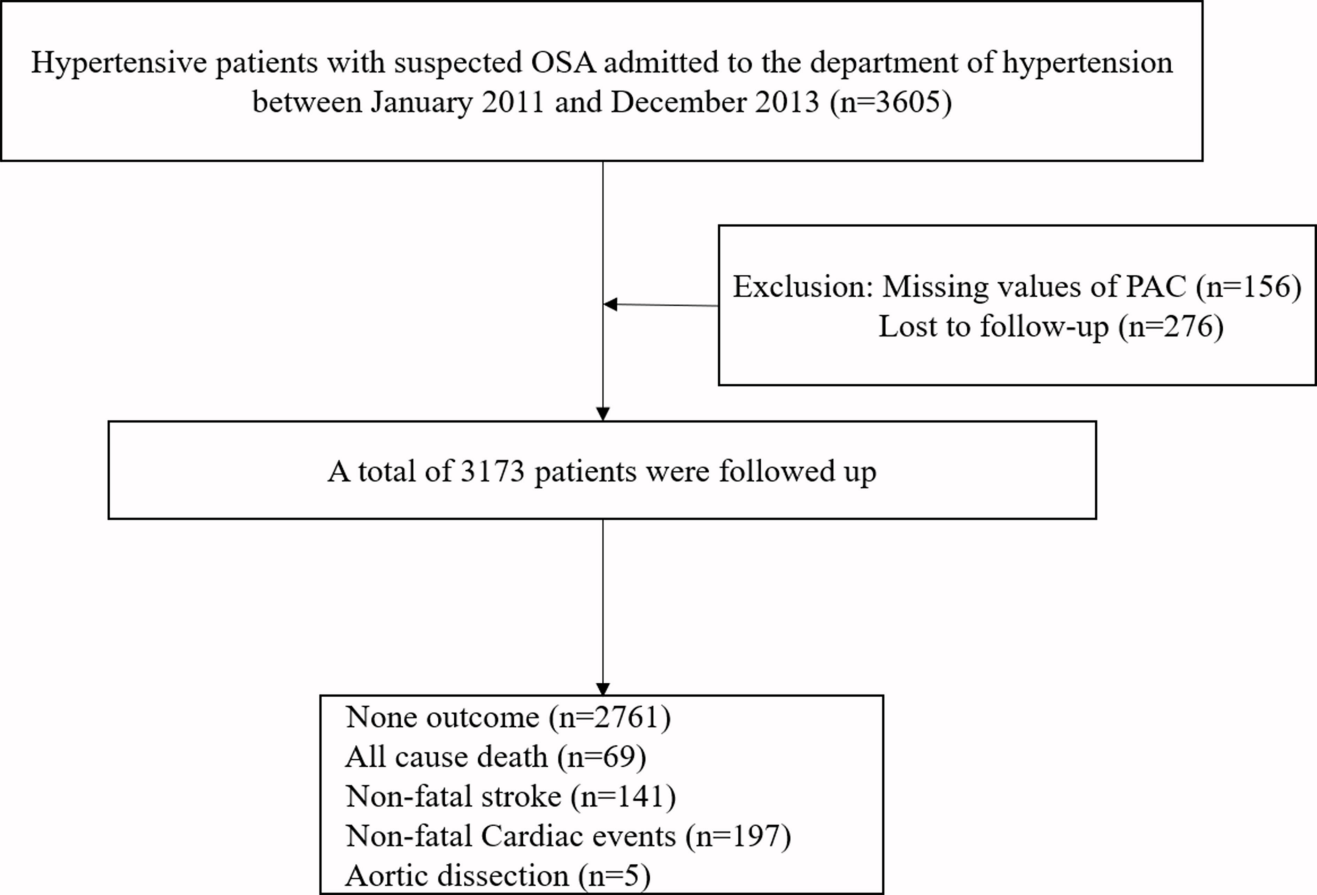
Flowchart of participants for this study.

Finally, 3173 individuals comprised analytical sample (**Figure 1**). The current study was approved by Ethics Committee of the People’s Hospital of Xinjiang Uygur Autonomous Region.

### Measurements of PAC and PRA

2169 (68.4%) hypertensives completed the measurement of PAC and PRA without interfering agents. That is, diuretics (including mineralocorticoid antagonists) were discontinued for at least 6 weeks, and dihydropyridine calcium antagonist, β-receptor blockers, angiotensin-converting enzyme inhibitors (ACEIs), and angiotensin receptor blockers (ARBs) were ceased for at least 4 weeks, or any antihypertensive medications were not taken at least 2 weeks before PAC and PRA measurement. When necessary, patients were allowed to take α-blockers (doxazosin or terazosin) and/or non-dihydropyridine calcium channel blocker (slow-release verapamil). 1004 (31.6%) participants completed the measurement of PAC and PRA under existence of interfering agents. All blood samples were collected in the morning after patients had been ambulant for at least 2h and rested for 15 min in sitting position. PAC was measured by radioimmunoassay using a commercially available kit (Beckman Coulter, Brea, CA, USA), the intra- and inter-assay coefficients of variation were 5.2 and 8.3, respectively. PRA was measured by using an iodine angiotensin I radioimmunoassay kit (Northern Biotechnology Institutes, Beijing, China), and the intra- and inter-assay coefficients of variation were 9.5 and 13.4, respectively. ARR was then calculated by dividing the PAC by PRA. The suspicious PA were defined as ARR≥20 (ng/dl)/(ng/ml per h) and PAC≥12ng/dl. The saline infusion test (SIT) was performed in suspect PA patients and the diagnosis of PA after SIT was based on Endocrine Society Guideline (24).

### Overnight sleep study and diagnosis of OSA

All patients underwent in-laboratory overnight PSG (Compumedics E series, Australia) examination. OSA was defined as an apnea-hypopnea index (AHI)≥5 events per hour; further, the severity of OSA was defined as follows: mild OSA (5≤AHI<15), moderate OSA (15≤AHI<30), and severe OSA (AHI≥30).

### Covariates

Other information as covariates, including age, gender, body mass index (BMI), waist circumference, cigarette consumption (yes/no), alcohol intake (yes/no), duration of hypertension, diabetes mellitus (DM, yes/no), apnea-hypopnea index (AHI), office systolic blood pressure (SBP), office diastolic blood pressure (DBP), uric acid (UA), serum creatinine (Scr), alanine aminotransferase (ALT), aspartate aminotransferase (AST), fasting plasma glucose (FPG), total cholesterol (TC), triglycerides (TG), high density lipoprotein cholesterol (HDL-C), low density lipoprotein cholesterol (LDL-C), lipoprotein a (Lp a), homocysteine (Hcy), serum potassium, serum sodium, and information on lipid regulating, antiplatelet and antihypertensive agents were collected as well.

Seated BP at hospitalization was measured in the upper arm after patients rested quietly for 10 mins at least with a mercury sphygmomanometer using international recommendations (25). The mean value of two measurements was recorded and used for analysis. BMI was measured in units of weight(kg)/height^2^(m). Estimated glomerular filtration rate(eGFR) was calculated using CKD-EPI equation (26).

### Follow up and outcomes

Composite outcome were incident CVD and all-cause mortality. CVD included non-fatal stroke (ischemic stroke, hemorrhagic stroke, transient ischemic attack), coronary events (acute myocardial infarction, coronary revascularization, hospitalized unstable angina), hospitalized heart failure, and aortic dissection. All participants were followed up through medical records, outpatient and or inpatient visits, and telephone communication. All events were certified by medical documents and confirmed by the clinical event committee. The deadline for follow-up was Jan 2021.

### Statistical analyses

Continuous variables with a normal distribution were presented as mean ± standard deviation and compared between groups using analysis of variance (ANOVA). Non-normally distributed continuous variables were presented as median (interquartile range) and compared between groups using Kruskal-Wallis H test. Categorical variables were expressed as frequency and percentage, and chi-square test was used for comparison between groups.

Kaplan-Meier curve was used to evaluate the relationship of PAC, PRA, and ARR with the composite outcomes, and log-rank test was used for comparison. To evaluate the validity of the proportional hazard assumption, the assumption was evaluated using log-minus-log-survival function and was found valid. Cox proportional hazard regression models were used to evaluate hazard ratios (HR) and 95% confidence intervals (CI) with inclusion of PAC as categorical (tertiles) or continuous variables (per 1, 5, 10 unit). Based on least absolute shrinkage and selection operator (LASSO) regression and univariate COX regression analysis, multivariate modeling was performed through the following sequential adjustments:

Model 1 (based on LASSO regression) was adjusted for age, DM at baseline, duration of hypertension≥5 years, cigarette consumption, alcohol intake, waist circumference, SBP, DBP, eGFR, TG, HDL-C, Lp(a), serum potassium, and PRA;

Model 2 (based on univariate COX regression analysis) was adjusted for gender, age, BMI, DM at baseline, duration of hypertension≥5 years, cigarette consumption, alcohol intake, AHI≥15 event per hour, waist circumference, SBP, DBP, eGFR, TC, TG, HDL-C, LDL-C, Lp(a), serum potassium, and PRA;

Model 3 was Model 2 plus antihypertensive agents, statins, antiplatelet agents, and regular CPAP treatment.

Considering that composite outcomes would change over time, we used time-dependent ROC curve with highest Youden index to determine the cut-off for PAC. Additionally, to identify potential effects of variables on the relationship between PAC and composite outcomes, stratified analyses were further performed according to gender, age (≤55 or >55 years), cigarette consumption (yes or no), alcohol intake (yes or no), BMI (<28 or ≥28kg/m^2^), AHI (<15 or ≥15 event per hour), SBP (<135 or ≥135mmHg), DBP (<85 or≥85mmHg), PRA (≤0.5 or >0.5ng/ml per h), and interfering agents (yes or no). We performed sensitivity analyses to further evaluate whether the association was present and stable by excluding patients with follow-up time less than 12 months, and with CVD at baseline. In view of the positive association between PA and CVD, we performed sensitivity analyses after exclusion of participants with suspicious PA (defined as ARR≥20 and PAC≥12), and with diagnosed PA. Furthermore, considering the potential influence of renal dysfunction on PAC and composite outcomes, we conducted a sensitivity analysis after exclusion of participants with eGFR<60 (ml/min per 1.73m^2^) at baseline. Statistical analyses were conducted in SPSS version 25.0 for Windows (SPSS Inc., Chicago, Illinois, USA) and R version 4.0.5.

## Results

### Baseline characteristics

A total of 3173 hypertensives were included, aged 48.2 ± 10.8 years. Among them, 2097(66.1%) were men, 1066(33.1%) were cigarette consumers, and 1051(33.1%) were alcohol takers. Median (interquartile range) was 13.1(9.0,19.1) ng/dl for PAC, 1.4(0.6,2.8) ng/ml/h for PRA, and 9.8(4.9,23.2) ng/dl per ng/ml/h for ARR. Mean SBP and DBP were 139.8 ± 19.5mmHg and 92.1 ± 13.9mmHg, respectively.

Participants were divided into three groups as T1 (<10.35), T2 (10.35–16.69) and T3 (>16.69) using tertiles of PAC at baseline. Participants in the third tertile of PAC were more likely to be younger, and had higher SBP and DBP. Additionally, participants with higher PAC had lower eGFR, HDL-C and serum potassium. Details of participant characteristics by tertiles of PAC at baseline were shown in **Table 1**.

**Table 1:** Baseline characteristics of groups classified by PAC tertiles.

	PAC tertiles (ng/dl)			P value
	<10.35 (n=1058)	10.35-16.69 (n=1057)	>16.69 (n=1058)	
Male, n (%)	689 (65.1)	715 (67.6)	693 (65.5)	P=0.418
Age (year)	49.5 ± 10.9	47.8 ± 10.8	47.3 ± 10.5	P<0.001
BMI (kg/m^2^)	27.9 ± 3.9	28.1 ± 3.8	27.9 ± 3.8	P=0.218
Waist circumference (cm)	99 (92,106)	100 (93,106)	99 (92,105)	P=0.199
Cigarette consumption (yes, n %)	368 (34.8)	344 (32.5)	354 (33.5)	P=0.549
Alcohol intake (yes, n %)	354 (33.5)	346 (32.7)	351 (33.2)	P=0.938
Hypertension Duration (≥5 years, n %)	440 (41.6)	452 (42.8)	450 (42.5)	P=0.845
DM at baseline, n (%)	167 (15.8)	173 (16.4)	165 (15.6)	P=0.880
AHI ≥15 per h, n (%)	448 (32.3)	470 (33.9)	467 (33.7)	P=0.570
Systolic blood pressure (mmHg)	138.5 ± 19.1	139.9 ± 18.8	141.0 ± 20.5	P=0.013
Diastolic blood pressure (mmHg)	90.7 ± 13.8	92.3 ± 13.4	93.2 ± 14.2	P<0.001
eGFR (ml/min per 1.73m^2^)	96.6 ± 17.9	95.7 ± 16.7	93.2 ± 20.4	P<0.001
Uric acid (μmol/L)	341.6 ± 91.6	344.4 ± 90.5	348.7 ± 97.9	P=0.215
Alanine aminotransferase (U/L)	26.9 ± 19.7	28.3 ± 18.8	30.0 ± 27.3	P=0.007
Aspartate aminotransferase (U/L)	22.1 ± 13.2	22.1 ± 16.3	22.4 ± 13.0	P=0.897
Fast food glucose (mmol/L)	4.9 (4.5,5.4)	5.0 (4.5,5.5)	4.9 (4.5,5.5)	P=0.331
Total cholesterol (mmol/L)	4.55 ± 1.15	4.49 ± 1.03	4.49 ± 1.08	P=0.392
Triglyceride (mmol/L)	2.09 ± 1.71	2.12 ± 1.71	2.13 ± 1.57	P=0.840
HDL-C (mmol/L)	1.13 ± 0.29	1.12 ± 0.29	1.10 ± 0.29	P=0.046
LDL-C (mmol/L)	2.63 ± 0.80	2.61 ± 0.81	2.59 ± 0.76	P=0.465
Lp (a) (mg/L)	156.0 (110.7,216.0)	155.0 (112.0,214.0)	154.0 (113.0,217.0)	P=0.947
Homocysteine (μmol/L)	15.8 (11.0,19.7)	16.2 (11.3,20.0)	16.5 (11.4,20.3)	P=0.786
Serum potassium (mmol/L)	3.94 ± 0.33	3.93 ± 0.32	3.88 ± 0.32	P<0.001
Serum sodium (mmol/L)	140.8 ± 2.6	140.8 ± 2.6	140.9 ± 2.5	P=0.657
PAC (ng/dl)	7.7 (6.3,9.0)	13.1 (11.7,14.8)	22.0 (19.1,27.5)	P<0.001
PRA (ng/ml per h)	0.9 (0.4,2.0)	1.3 (0.6,2.6)	2.1 (0.9,4.1)	P<0.001
ARR (ng/dl)/ (ng/ml per h)	7.9 (3.7,20.0)	9.8 (5.0,21.9)	11.1 (6.0,26.6)	P<0.001
Medications on Discharge, n (%)				
ACEI/ARB	559 (52.8)	527 (49.9)	478 (45.2)	P=0.002
β-blocker	112 (10.6)	97 (9.2)	110 (10.4)	P=0.505
CCB	738 (68.8)	771 (72.9)	796 (75.2)	P=0.018
Diuretic	152 (14.4)	163 (15.4)	201 (19.0)	P=0.010
Statins	559 (52.8)	525 (49.7)	526 (49.7)	P=0.248
Antiplatelet agents	530 (50.1)	506 (47.9)	502 (47.4)	P=0.425
Regular CPAP treatment, n (%)	38 (3.6)	33 (3.1)	31 (2.9)	P=0.674

AHI, Apnea-Hypopnea Index; ACEI, angiotensin-converting-enzyme inhibitors; ARB, angiotensin receptor blockers; eGFR, estimated glomerular filtration rate; HDL-C, high density lipoprotein cholesterol; LDL-C, low density lipoprotein cholesterol; PAC, plasma aldosterone concentration; PRA, plasma renin activity; ARR, aldosterone to renin activity ratio; CPAP, continuous positive airway pressure.

As shown in **Table 2**, during a median follow-up period of 7.3 years (interquartile range, 6.4-8.2) and 22640.25 person-years, 69 deaths and 343 incident CVD occurred with incidence of 18.2/1000 person-years in all participants. Incidence in third tertile was 23.9/1000 person-years.

**Table 2:** Follow-up time and incidence of primary outcomes in total patients and tertiled by plasma aldosterone concentration.

	Total (n=3173)	T1 (n=1058)	T2 (n=1057)	T3 (n=1058)
Follow-up time, median	7.3 (6.4-8.2)	7.3 (6.5-8.2)	7.3 (6.5-8.2)	7.3 (6.4-8.2)
Person-years followed	22640.25	7604.08	7598.92	7437.25
Outcomes per 1000 person years	18.2	12.9	17.9	23.9

### Association of PAC and composite outcomes

**Figure 2:**
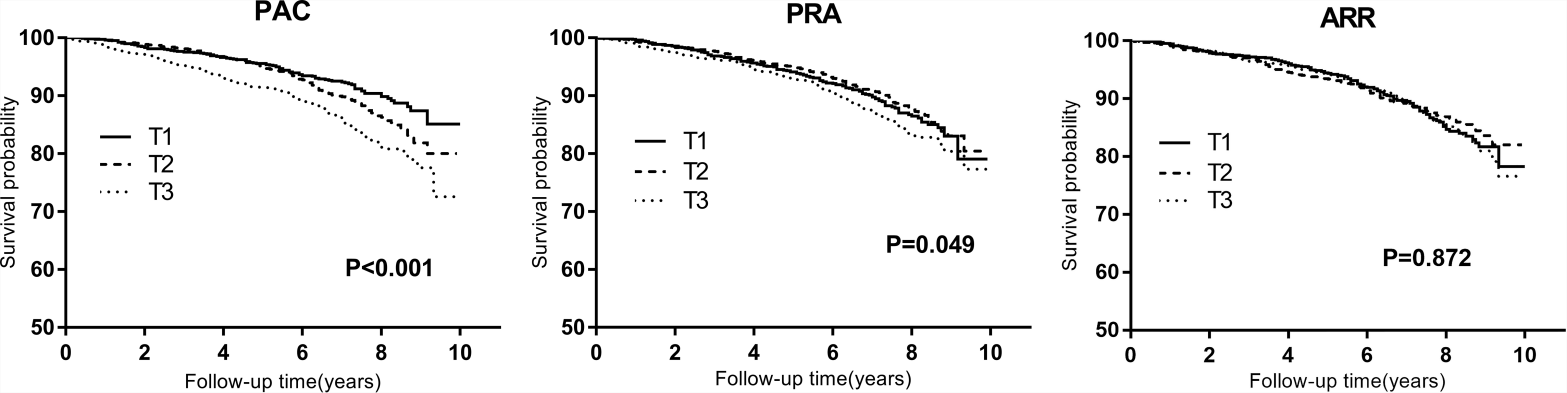
Kaplan-Meier curves for composite outcomes across tertiles of plasma aldosterone concentration (PAC), plasma renin activity (PRA), and aldosterone-to-renin ratio (ARR). T1 to T3 indicate ascending tertiles; T1 below 10.35(n=1058), T2 10.35–16.69 (n=1057), T3 above 16.69 (ng/dl) (n=1058) for PAC; T1 below 0.80(n=1055), T2 0.80–2.16 (n=1064), T3 above2.16 (ng/ml per h) (n=1054) for PRA; T1 below 6.05(n=1057), T2 6.05–16.33 (n=1060), T3 above 16.33(ng/dl)/(ng/ml per h) (n=1056) for ARR.

Kaplan-Meier curves for composite outcomes according to PAC, PRA, and ARR tertiles were presented in **Figure 2**. The curve depicted an increased risk of composite outcomes in participants in the top tertile of PAC (P log-rank<0.001).

Cox regression showed that high PAC was associated with increased risk of CVD and all-cause death in unadjusted model, with HR of 1.86 (T3 vs T1:HR=1.86, 95%CI, 1.45-2.38, P<0.001), which remained significant in full adjusted model (**Table 3**). Compared with patients in first tertile, adjusted HR was 1.81 for those in third tertile (95%CI,1.39-2.35, P<0.001). Every 1, 5 and 10 unit increase of PAC was associated with a 1%, 7%, and 14% higher risk of CVD and all-cause death, respectively, in full adjusted model (Model3). Sensitivity analyses by excluding those with follow-up less than 12 months, reduced eGFR (<60 ml/min per 1.73m^2^), those with CVD at baseline, suspicious PA patients, or diagnosed PA patients did not change the association between PAC and composite outcomes (**Tables S2** through **S6**).

**Table 3:** Association of plasma aldosterone concentration with risk of composite outcomes.

PAC	Crude model	Model 1	Model 2	Model 3
	HR (95%CI) P value	HR (95%CI) P value	HR (95%CI) P value	HR (95%CI) P value
Tertile 1	Reference	Reference	Reference	Reference
Tertile 2	1.35 (1.04-1.75) 0.027	1.33 (1.02-1.74) 0.033	1.33 (1.02-1.73) 0.037	1.36 (1.04-1.78) 0.023
Tertile 3	1.86 (1.45-2.38) < 0.001	1.76 (1.36-2.29) < 0.001	1.76 (1.36-2.28) < 0.001	1.81 (1.39-2.35) < 0.001
P for trend	< 0.001	< 0.001	< 0.001	< 0.001
PAC+1ng/dl	1.02 (1.01-1.03) < 0.001	1.02 (1.01-1.03) 0.016	1.01 (1.01-1.02) 0.019	1.01 (1.00-1.02) 0.009
PAC+5ng/dl	1.10 (1.05-1.15) < 0.001	1.06 (1.02-1.11) 0.016	1.06 (1.01-1.11) 0.019	1.07 (1.02-1.12) 0.009
PAC+10ng/dl	1.20 (1.10-1.32) < 0.001	1.13 (1.02-1.24) 0.016	1.12 (1.02-1.23) 0.019	1.14 (1.03-1.25) 0.009

Model 1: adjusted for age, T2DM at baseline, Duration of hypertension≥5 years, cigarette consumption, alcohol intake, waist circumference, SBP, DBP, eGFR, TG, HDL-C, Lp(a), serum potassium, PRA; Model 2: adjusted for gender, age, BMI, T2DM at baseline, Duration of hypertension≥5 years, cigarette consumption, alcohol intake, AHI≥15, waist circumference, SBP, DBP, eGFR, TC, TG, HDL-C, LDL-C, Lp(a), serum potassium, PRA; Model 3: Model2+ antihypertensive agents, statins, antiplatelet agents, regular CPAP treatment.

Time-dependent ROC curve with the highest Youden index was used to determine the optimal cut-off value of PAC for predicting 8-year CVD and all-cause death, then we determined that the optimal threshold for PAC was 12.5ng/dl (AUC=0.573, 95%CI: 0.539-0.607; +LR=1.401, -LR=0.632; Se=0.669, Sp=0.524).

We divided all patients into two groups using the observed cut-off as <12.5ng/dl and ≥12.5ng/dl groups. In binary variables, the incidence of composite outcomes in the patients with PAC ≥12.5ng/dl was significantly higher than those with PAC <12.5ng/dl, and HRs for unadjusted model and full adjusted model 3 were 1.65 (95%CI,1.34-2.03, P<0.001), and 1.61 (95%CI, 1.30-1.99 P<0.001), respectively.

### Association of PAC and composite outcomes in stratification

**Figure 3:**
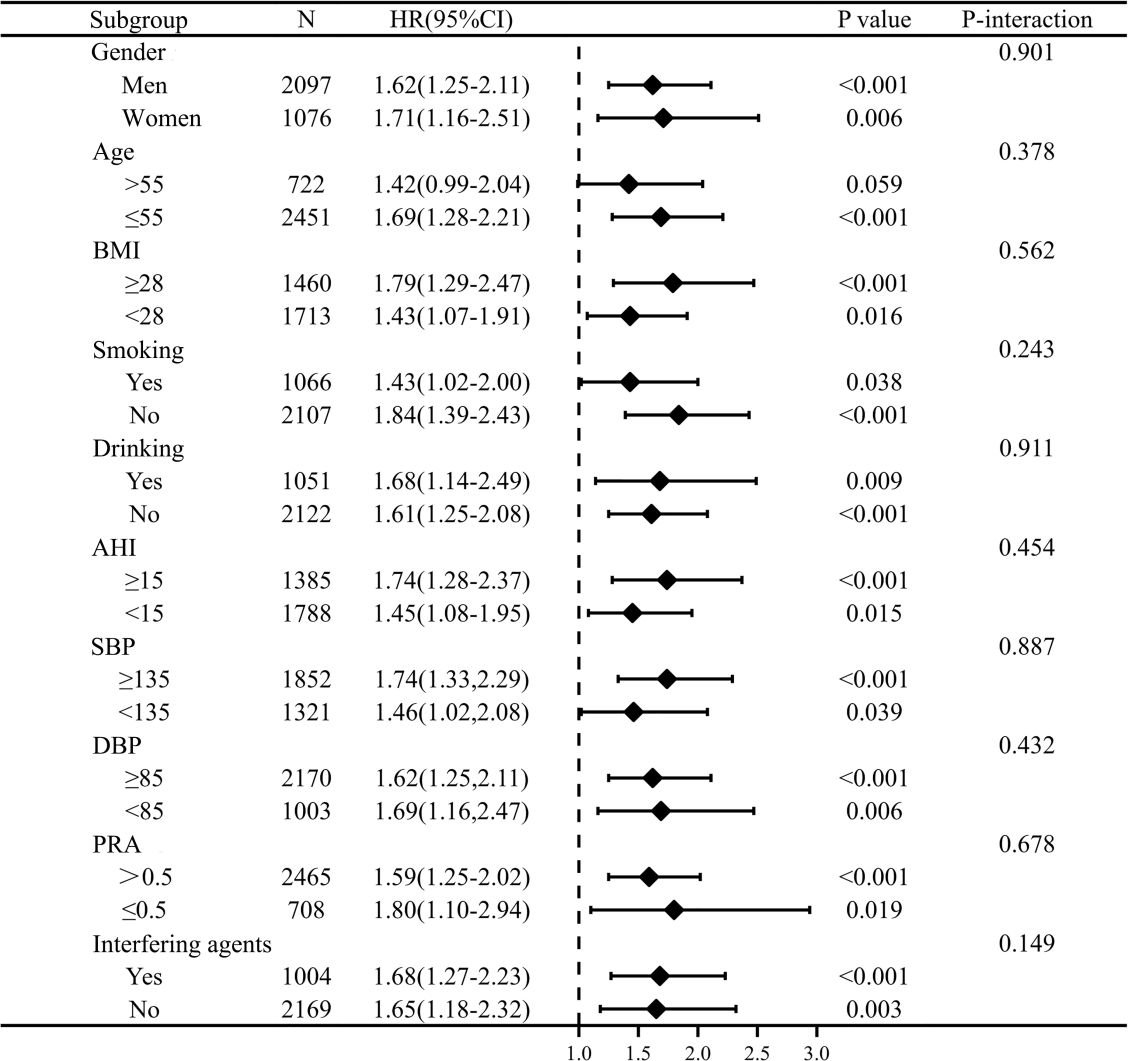
Stratification analysis on relationship of PAC with outcome. PAC was converted to binary variables according to the cut-off value of 12.5ng/dl *via* time-dependent receiver operating characteristic curve. The model was adjusted for gender, age, BMI, DM at baseline, duration of hypertension≥5, cigarette consumption, alcohol intake, AHI≥15, waist circumference, SBP, DBP, eGFR, TC, TG, HDL-C, LDL-C, Lp(a), serum potassium, PRA, and antihypertensive agents, statins, antiplatelet agents, regular CPAP treatment.

Stratified analyses were performed to evaluate the association of PAC as a binary variable (≥12.5 versus <12.5ng/dl) with the risk of incident composite outcomes. Gender, age, cigarette consumption, alcohol intake, BMI, AHI, SBP, DBP, PRA, and interfering agents on PAC had no significant effect modification on the PAC composite outcomes association (P for interaction>0.05) (**Figure 3**). Whether renin is suppressed or not, elevated PAC is independently associated with increased CVD risk.

## Discussion

Recent studies have focused on deleterious effects of circulating aldosterone on cardiovascular morbidity and mortality in various populations with inconsistent results among studies and with few data in hypertensives, a group of population with elevated risk for CVD and a huge burden worldwide due to increasing prevalence. To our knowledge, this is the first study in this specific population with inclusion of conditions like OSA and PA. The main observations of the current retrospective cohort study encompassed the following. Higher PAC is associated with higher risks of CVD and all-cause death in hypertensives, independent of established CVD risk factors, of antihypertensive, antiplatelet and lipid regulating treatments, of PRA and even independent of OSA and PA. The optimal cut-off value of PAC for predicting the occurrence of CVD is 12.5ng/dl.

Recently, with recommendation and promotion of screening for PA in hypertensives, damage of PAC to cardiovascular system have gradually acquired more attention. Patients with PA are more likely to develop CVD than patients with essential hypertension, in which PAC may play important roles (7, 8, 27). Current results of UROSAH add some evidence for ongoing debate and or uncertainty and extend previous findings to hypertensive patients. That is, higher PAC is associated with increased risk of CVD and all-cause mortality. Consistent with current observations, LURIC study found that higher PAC is associated with increased CVD and all-cause mortality in elderly patients referred to coronary angiography (17); OPERA study also observed higher PAC is associated with the occurrence of death, heart failure, and acute renal failure in patients with acute myocardial infarction (28). Nonetheless, considering that these results were derived from patients with cardiac insufficiency or ACS in the above studies, it is unclear whether PAC directly damages cardiovascular system, or it is just a marker of RAAS overactivation, and catecholamine surges. Another study from community dwelling African Americans, which included 60% of hypertensive patients, reported that elevated PAC may play important roles in developing CVD and all-cause death (18). Nonetheless, differences in PAC levels among ethnic or racial entities also affect the stability and extrapolation of the results.

Some previous findings have suggested that PAC were associated with cardiac structural and increased risk of all-cause mortality in renin-suppressed group (15, 29). Our stratified analysis shows whether renin is suppressed or not (≤0.5 or >0.5ng/ml per h), elevated PAC is independently associated with an increased risk of CVD. Similarly, sensitivity analysis by exclusion of suspicious PA or diagnosed PA did not change the association between PAC and CVD. These findings imply that attention should still be paid to the harm caused by elevated PAC in hypertensive patients in the absence of PA. Considering the interfering effects of medications on PAC and PRA measurement (24), we performed analysis by presence of interfering agents (yes or no) at PAC measurement and observed that it had no effect on the results (P for interaction=0.149). Considering the impact of PA and of interfering medications on PAC measurement is an important part of our study, since most previous studies failed to analyze. This may also be important for the generalization of current results to patients with essential hypertension. Furthermore, the age stratified analysis found that there was a weakened association between PAC and composite outcomes in the subgroup of age >55 years (30), which may be related to the small number of participants in this group(n=722), whereas further studies are needed.

We conducted this study in hypertensive patients with suspected OSA, and the bidirectional relationship between aldosterone and OSA (31, 32) may limit the generalizability of current results to general hypertensive population. However, hypertension and OSA commonly co-exist. Prevalence of OSA among hypertensive patients is estimated to be about 30%-50% (33), and it is still increasing worldwide (34). In addition, to eliminate the influence of OSA, AHI was taken into account as one of the confounders in Cox regression. Furthermore, stratified analysis by presence of OSA still showed elevated PAC is still a risk factor for CVD in hypertensives with and without OSA.

Currently, few studies have explored the cut-off value of PAC to determine hypertensive population at high risk of developing CVD. A Japanese retrospective cross-sectional study of PA patients observed that patients with PAC≥125pg/ml show a significantly increased risk of CVD and recommended PA-specific treatments to these patients (27). We believe that it is necessary to calculate the optimal cut-off value, which may be helpful for guiding the treatment and management strategy of hypertensive patients. Due to aldosterone escape, interruption of RAAS by ACEI and ARB may not be able to attenuate harmful effects of aldosterone (23). Previous studies have found that mineralocorticoid receptor antagonists (MRAs) are effective in improving the prognosis of patients with heart failure and treating resistant hypertension (35–37), and are also a key treatment for PA patients. Whether to use MRAs to antagonize damage of PAC is a question worth pondering for hypertensive patients with PAC≥12.5ng/dl, at least in Chinese population based on current observation, whereas more studies are warranted.

The current study has several strengths. First, we evaluated the relationship between PAC and cardiovascular events in larger-scale hypertensive patients through longitudinal follow-up. Second, we considered the potential effects of the antihypertensive, antiplatelet and lipid regulating agents, renin, and OSA in the Cox regression. Third, we considered the potential influence of OSA, suspicious PA or diagnosed PA in stratified analyses and sensitivity analyses. Fourth, we evaluated the impact of interfering agents at PAC measurement on the results, which was not accomplished in other studies. Nevertheless, several limitations need to be discussed. First, we lacked 24-hour urine sodium data to assess whether sodium intake had an impact on the relationship between PAC and CVD. Second, we calculated cut-off value of PAC, above which CVD risk was increased. However, the extrapolation of the cut-off value may be affected by the study population and region. Third, we also lacked data on changes in medications, lifestyle and PAC levels during follow-up period, which might affect our results. Fourth, the different detection methods of aldosterone concentration are not consistent. Compared with LC/MS method, radioimmunoassay has errors for detection of lower concentrations of aldosterone, most probably from cross reactivity with soluble aldosterone metabolite, which may limit the extrapolation of our results.

In conclusion, higher PAC is associated with increased risk of CVD and all-cause mortality, even in the absence of PA. Hypertensive patients with PAC≥12.5ng/dl have a significantly increased risk of CVD and special attention and treatment need to be considered.

## Data availability statement

The raw data supporting the conclusions of this article will be made available by the authors, without undue reservation.

## Ethics statement

The studies involving human participants were reviewed and approved by Ethics Committee of the People’s Hospital of Xinjiang Uygur Autonomous Region. The patients/participants provided their written informed consent to participate in this study.

## Author contributions

LG contributed to the study design and statistical analysis; LG, NL, MH, ML and QZ analyzed the data together and drafted the manuscript; XY, TW, MW, QL, DZ, WJ, and JH participated in data collection. All authors have read and approval the final manuscript.

## Funding

This research was supported by the Non-profit Central Research Institute Fund of Chinese Academy of Medical Sciences (grant number: 2020-RW330-002).

## Acknowledgments

We thank all participants and staff of the UROSAH study for their important contributions.

## Conflict of interest

The authors declare that the research was conducted in the absence of any commercial or financial relationships that could be construed as a potential conflict of interest.

## Publisher’s note

All claims expressed in this article are solely those of the authors and do not necessarily represent those of their affiliated organizations, or those of the publisher, the editors and the reviewers. Any product that may be evaluated in this article, or claim that may be made by its manufacturer, is not guaranteed or endorsed by the publisher.
