# Supplementary material for: Higher plasma aldosterone is associated with increased risk of cardiovascular events in hypertensive patients with suspected OSA: UROSAH data
Source: Front Endocrinol (Lausanne). 2022 Oct 7;13:1017177. doi: 10.3389/fendo.2022.1017177 (PMC9585258; doi:10.3389/fendo.2022.1017177)
Supplement: Supplementary file 1 [file DataSheet_1.docx]

**SUPPLEMENTARY MATERIAL**

**Higher Plasma Aldosterone Is Associated with Increased Risk of** **Cardiovascular Events in Hypertensive Patients with Suspected OSA: UROSAH Data**

**All author’s names:** Lin Gan^1^, Nanfang Li^1^, Mulalibieke Heizati^1^, Mengyue Lin^1^, Qing Zhu^1^, Xiaoguang Yao^1^, Ting Wu^1^, Menghui Wang^1^, Qin Luo^1^, Delian Zhang^1^, Wen Jiang^1^, Junli Hu^1^

**Authors’ affiliations:**

^1^Hypertension Center of People’s Hospital of Xinjiang Uygur Autonomous Region; Xinjiang Hypertension Institute; National Health Committee Key Laboratory of Hypertension Clinical Research; Key Laboratory of Xinjiang Uygur Autonomous Region “Hypertension Research Laboratory; Xinjiang Clinical Medical Research Center for Hypertension (Cardio-Cerebrovascular) Diseases.

**Corresponding author:** Professor Nanfang Li, Hypertension Center of People’s Hospital of Xinjiang Uygur Autonomous Region, 91 Tianchi Road, Urumqi, Xinjiang 830001, China. E-mail: lnanfang2016@sina.com

| Table S1. Univariate COX regression analysis and colinearity diagnosis for variables. | | | | | |
| --- | --- | --- | --- | --- | --- |
| Variable | P | HR | 95% CI | Tolerance | VIF |
| Age | <0.001 | 1.03 | 1.02-1.03 | 0.527 | 1.897 |
| Gender | 0.201 | 1.15 | 0.93-1.42 | 0.565 | 1.769 |
| BMI | 0.014 | 1.03 | 1.01-1.06 | 0.334 | 2.998 |
| Waist circumference | <0.001 | 1.02 | 1.01-1.03 | 0.320 | 3.121 |
| Cigarette consumption | 0.004 | 1.34 | 1.09-1.63 | 0.680 | 1.470 |
| Alcohol intake | 0.118 | 0.84 | 0.68-1.04 | 0.690 | 1.448 |
| Hypertension Duration (≥5 years) | <0.001 | 1.59 | 1.31-1.94 | 0.840 | 1.191 |
| DM at baseline | <0.001 | 1.77 | 1.41-2.23 | 0.899 | 1.112 |
| AHI ≥15 per h | 0.003 | 1.34 | 1.10-1.63 | 0.863 | 1.159 |
| Systolic blood pressure | <0.001 | 1.01 | 1.01-1.02 | 0.451 | 2.219 |
| Diastolic blood pressure | 0.012 | 1.01 | 1.01-1.02 | 0.419 | 2.385 |
| eGFR | <0.001 | 0.99 | 0.99-1.00 | 0.747 | 1.338 |
| TC | 0.372 | 1.04 | 0.95-1.13 | 0.400 | 2.497 |
| TG | 0.009 | 1.06 | 1.02-1.11 | 0.653 | 1.532 |
| HDL-C | 0.079 | 0.73 | 0.52-1.04 | 0.805 | 1.242 |
| LDL-C | 0.645 | 1.03 | 0.91-1.16 | 0.484 | 2.065 |
| Lp(a) | 0.001 | 1.00 | 1.00-1.01 | 0.982 | 1.018 |
| Serum potassium | 0.040 | 0.73 | 0.54-0.99 | 0.935 | 1.069 |
| PRA | <0.001 | 1.07 | 1.03-1.11 | 0.894 | 1.119 |
| Medications |  |  |  |  |  |
| ACEI/ARB | 0.007 | 1.31 | 1.08-1.59 | 0.899 | 1.112 |
| β-blocker | <0.001 | 1.71 | 1.30-2.25 | 0.930 | 1.075 |
| CCB | <0.001 | 1.62 | 1.27-2.07 | 0.858 | 1.166 |
| Diuretic | 0.024 | 1.33 | 1.04-1.70 | 0.907 | 1.102 |
| Statins | <0.001 | 1.94 | 1.58-2.37 | 0.584 | 1.712 |
| Antiplatelet | <0.001 | 1.76 | 1.44-2.14 | 0.586 | 1.706 |
| Regular CPAP treatment | 0.742 | 1.09 | 0.64-1.86 | 0.970 | 1.031 |

AHI, Apnea-Hypopnea Index; ACEI, angiotensin-converting-enzyme inhibitors; ARB, angiotensin receptor blockers; eGFR, estimated glomerular filtration rate; HDL-C, high density lipoprotein cholesterol; LDL-C, low density lipoprotein cholesterol; PAC, plasma aldosterone concentration; PRA, plasma renin activity; ARR, aldosterone to renin activity ratio; CPAP, continuous positive airway pressure.

| Table S2. Sensitivity analysis excluding subjects with incident CVD≤1 year (n=22). | | | | |
| --- | --- | --- | --- | --- |
| PAC | Crude model | Model 1 | Model 2 | Model 3 |
|  | HR (95%CI) P value | HR (95%CI) P value | HR (95%CI) P value | HR (95%CI) P value |
| Tertile 1 | (ref) | (ref) | (ref) | (ref) |
| Tertile 2 | 1.34(1.02-1.75) 0.034 | 1.34(1.02-1.75) 0.036 | 1.32(1.01-1.74) 0.043 | 1.36(1.03-1.78) 0.029 |
| Tertile 3 | 1.79(1.39-2.31) <0.001 | 1.72(1.32-2.25) <0.001 | 1.71(1.31-2.24) <0.001 | 1.75(1.34-2.29) <0.001 |
| P for trend | <0.001 | <0.001 | <0.001 | <0.001 |
| PAC+1ng/dl | 1.02(1.01-1.03) 0.001 | 1.01(1.00-1.03) 0.044 | 1.01(1.00-1.02) 0.052 | 1.01(1.00-1.02) 0.030 |
| PAC+5ng/dl | 1.09(1.04-1.14) 0.001 | 1.05(1.01-1.10) 0.044 | 1.05(1.00-1.11) 0.052 | 1.06(1.01-1.11) 0.030 |
| PAC+10ng/dl | 1.18(1.07-1.30) 0.001 | 1.10(1.01-1.23) 0.044 | 1.11(1.00-1.22) 0.052 | 1.12(1.01-1.24) 0.030 |

Model1: adjusted for age, T2DM at baseline, Duration of hypertension≥5 years, cigarette consumption, alcohol intake, waist circumference, SBP, DBP, eGFR, TG, HDL-C, Lp(a), serum potassium, PRA; Model2: adjusted for gender, age, BMI, DM at baseline, Duration of hypertension≥5 years, cigarette consumption, alcohol intake, AHI≥15, waist circumference, SBP, DBP, eGFR, TC, TG, HDL-C, LDL-C, Lp(a), serum potassium, PRA; Model3: Model2+ antihypertensive agents, statins, antiplatelet agents, regular CPAP treatment.

| Table S3. Sensitivity analysis excluding subjects with eGFR<60 (ml/min per 1.73m^2^) at baseline (n=149). | | | | |
| --- | --- | --- | --- | --- |
| PAC | Crude model | Model 1 | Model 2 | Model 3 |
|  | HR (95%CI) P value | HR (95%CI) P value | HR (95%CI) P value | HR (95%CI) P value |
| Tertile 1 | (ref) | (ref) | (ref) | (ref) |
| Tertile 2 | 1.33(1.01-1.74) 0.042 | 1.33(1.1-1.75) 0.040 | 1.33(1.01-1.75) 0.044 | 1.36(1.04-1.80) 0.028 |
| Tertile 3 | 1.88(1.45-2.42) <0.001 | 1.82(1.39-2.37) <0.001 | 1.81(1.39-2.36) <0.001 | 1.86(1.42-2.43) <0.001 |
| P for trend | <0.001 | <0.001 | <0.001 | <0.001 |
| PAC+1ng/dl | 1.02(1.01-1.03) <0.001 | 1.01(1.00-1.03) 0.010 | 1.01(1.00-1.02) 0.012 | 1.02(1.00-1.03) 0.005 |
| PAC+5ng/dl | 1.10(1.05-1.16) <0.001 | 1.07(1.02-1.13) 0.010 | 1.07(1.02-1.13) 0.012 | 1.08(1.02-1.14) 0.005 |
| PAC+10ng/dl | 1.21(1.10-1.33) <0.001 | 1.15(1.03-1.28) 0.010 | 1.15(1.03-1.27) 0.012 | 1.16(1.05-1.30) 0.005 |

Model1: adjusted for age, T2DM at baseline, Duration of hypertension≥5 years, cigarette consumption, alcohol intake, waist circumference, SBP, DBP, eGFR, TG, HDL-C, Lp(a), serum potassium, PRA; Model2: adjusted for gender, age, BMI, DM at baseline, Duration of hypertension≥5 years, cigarette consumption, alcohol intake, AHI≥15, waist circumference, SBP, DBP, eGFR, TC, TG, HDL-C, LDL-C, Lp(a), serum potassium, PRA; Model3: Model2+ antihypertensive agents, statins, antiplatelet agents, regular CPAP treatment.

| Table S4. Sensitivity analysis excluding subjects with history of CVD (n=59). | | | | |
| --- | --- | --- | --- | --- |
| PAC | Crude model | Model 1 | Model 2 | Model 3 |
|  | HR (95%CI) P value | HR (95%CI) P value | HR (95%CI) P value | HR (95%CI) P value |
| Tertile 1 | (ref) | (ref) | (ref) | (ref) |
| Tertile 2 | 1.35(1.03-1.76) 0.029 | 1.33(1.01-1.74) 0.039 | 1.32(1.01-1.73) 0.043 | 1.35(1.03-1.77) 0.029 |
| Tertile 3 | 1.89(1.47-2.43) <0.001 | 1.79(1.37-2.32) <0.001 | 1.78(1.37-2.32) <0.001 | 1.82(1.39-2.36) <0.001 |
| P for trend | <0.001 | <0.001 | <0.001 | <0.001 |
| PAC+1ng/dl | 1.02(1.01-1.03) <0.001 | 1.01(1.00-1.02) 0.009 | 1.01(1.00-1.02) 0.010 | 1.01(1.00-1.02) 0.006 |
| PAC+5ng/dl | 1.11(1.06-1.16) <0.001 | 1.07(1.02-1.12) 0.009 | 1.07(1.02-1.12) 0.010 | 1.07(1.02-1.13) 0.006 |
| PAC+10ng/dl | 1.23(1.12-1.35) <0.001 | 1.14(1.03-1.26) 0.009 | 1.14(1.03-1.26) 0.010 | 1.15(1.04-1.27) 0.006 |

Model1: adjusted for age, T2DM at baseline, Duration of hypertension≥5 years, cigarette consumption, alcohol intake, waist circumference, SBP, DBP, eGFR, TG, HDL-C, Lp(a), serum potassium, PRA; Model2: adjusted for gender, age, BMI, DM at baseline, Duration of hypertension≥5 years, cigarette consumption, alcohol intake, AHI≥15, waist circumference, SBP, DBP, eGFR, TC, TG, HDL-C, LDL-C, Lp(a), serum potassium, PRA; Model3: Model2+ antihypertensive agents, statins, antiplatelet agents, regular CPAP treatment.

| Table S5. Sensitivity analysis excluding subjects with ARR≥20 and PAC≥12 (n=552). | | | | |
| --- | --- | --- | --- | --- |
| PAC | Crude model | Model 1 | Model 2 | Model 3 |
|  | HR (95%CI) P value | HR (95%CI) P value | HR (95%CI) P value | HR (95%CI) P value |
| Tertile 1 | (ref) | (ref) | (ref) | (ref) |
| Tertile 2 | 1.14(0.85-1.53) 0.386 | 1.13(0.84-1.52) 0.434 | 1.12(0.83-1.51) 0.475 | 1.11(0.82-1.49) 0.514 |
| Tertile 3 | 1.65(1.25-2.16) <0.001 | 1.54(1.14-2.08) 0.005 | 1.53(1.13-2.06) 0.006 | 1.53(1.13-2.07) 0.006 |
| P for trend | <0.001 | 0.004 | 0.005 | 0.005 |
| PAC+1ng/dl | 1.02(1.01-1.03) <0.001 | 1.01(1.00-1.03) 0.078 | 1.01(1.00-1.02) 0.090 | 1.01(1.00-1.03) 0.057 |
| PAC+5ng/dl | 1.11(1.05-1.18) <0.001 | 1.06(0.99-1.13) 0.078 | 1.06(0.99-1.13) 0.090 | 1.07(1.00-1.14) 0.057 |
| PAC+10ng/dl | 1.24(1.11-1.39) <0.001 | 1.12(0.99-1.28) 0.078 | 1.12(0.98-1.27) 0.090 | 1.14(1.00-1.29) 0.057 |

Model1: adjusted for age, T2DM at baseline, Duration of hypertension≥5 years, cigarette consumption, alcohol intake, waist circumference, SBP, DBP, eGFR, TG, HDL-C, Lp(a), serum potassium, PRA; Model2: adjusted for gender, age, BMI, DM at baseline, Duration of hypertension≥5 years, cigarette consumption, alcohol intake, AHI≥15, waist circumference, SBP, DBP, eGFR, TC, TG, HDL-C, LDL-C, Lp(a), serum potassium, PRA; Model3: Model2+ antihypertensive agents, statins, antiplatelet agents, regular CPAP treatment.

| Table S6. Sensitivity analysis excluding subjects with diagnosed PA (n=441). | | | | |
| --- | --- | --- | --- | --- |
| PAC | Crude model | Model 1 | Model 2 | Model 3 |
|  | HR (95%CI) P value | HR (95%CI) P value | HR (95%CI) P value | HR (95%CI) P value |
| Tertile 1 | (ref) | (ref) | (ref) | (ref) |
| Tertile 2 | 1.32(0.99-1.76) 0.058 | 1.31(0.98-1.75) 0.070 | 1.30(0.97-1.74) 0.079 | 1.33(0.99-1.77) 0.058 |
| Tertile 3 | 1.90(1.46-2.49) <0.001 | 1.76(1.32-2.33) <0.001 | 1.75(1.32-2.33) <0.001 | 1.81(1.36-2.40) <0.001 |
| P for trend | <0.001 | <0.001 | <0.001 | <0.001 |
| PAC+1ng/dl | 1.02(1.01-1.03) <0.001 | 1.01(1.00-1.03) 0.020 | 1.01(1.00-1.03) 0.020 | 1.02(1.00-1.03) 0.011 |
| PAC+5ng/dl | 1.12(1.06-1.18) <0.001 | 1.07(1.01-1.13) 0.020 | 1.07(1.01-1.13) 0.020 | 1.08(1.02-1.14) 0.011 |
| PAC+10ng/dl | 1.26(1.13-1.40) <0.001 | 1.15(1.02-1.29) 0.020 | 1.15(1.02-1.29) 0.020 | 1.16(1.03-1.31) 0.011 |

Model1: adjusted for age, T2DM at baseline, Duration of hypertension≥5 years, cigarette consumption, alcohol intake, waist circumference, SBP, DBP, eGFR, TG, HDL-C, Lp(a), serum potassium, PRA; Model2: adjusted for gender, age, BMI, DM at baseline, Duration of hypertension≥5 years, cigarette consumption, alcohol intake, AHI≥15, waist circumference, SBP, DBP, eGFR, TC, TG, HDL-C, LDL-C, Lp(a), serum potassium, PRA; Model3: Model2+ antihypertensive agents, statins, antiplatelet agents, regular CPAP treatment.
